# Supplementary material for: Electrical Stimulation Directs Formation of Perfused Vasculature in Engineered Tissues
Source: Adv Sci (Weinh). 2026 May 10;13(33):e18677. doi: 10.1002/advs.202518677 (PMC13271649; doi:10.1002/advs.202518677)
Supplement: Supplementary file 1 — Supporting File: advs74992‐sup‐0001‐SuppMat.docx. [file ADVS-13-e18677-s001.docx]

**Supplementary Information for:**

**Electrical stimulation directs formation of perfused vasculature in engineered tissues**

Katarzyna A. Grzelak^1,2^, Ashley D. Westerfield^1,2^, Vardhman Kumar^2^, Kasturi Chakraborty^2,6^, Navaneeth Krishna Rajeeva Pandian^5^, Christopher S. Chen^3-5^, Sangeeta N. Bhatia^1-2, 5-6*^

^1^ Harvard-MIT Health Sciences and Technology, Institute for Medical Engineering and Science, Massachusetts Institute of Technology, Cambridge, MA 02139, USA

^2^ David H. Koch Institute for Integrative Cancer Research, Massachusetts Institute of Technology, Cambridge, MA, 02139 USA

^3^ Department of Bioengineering, Boston University, Boston, MA, 02215 USA

^4^ Biological Design Center, Department of Biomedical Engineering, Boston University, Boston, MA 02215, USA

^5^ Wyss Institute for Biologically Inspired Engineering at Harvard University, Boston, MA 02215, USA

^6^ Howard Hughes Medical Institute, Chevy Chase, MD 20815, US

*corresponding author email: sbhatia@mit.edu

***Supplemental Figures***


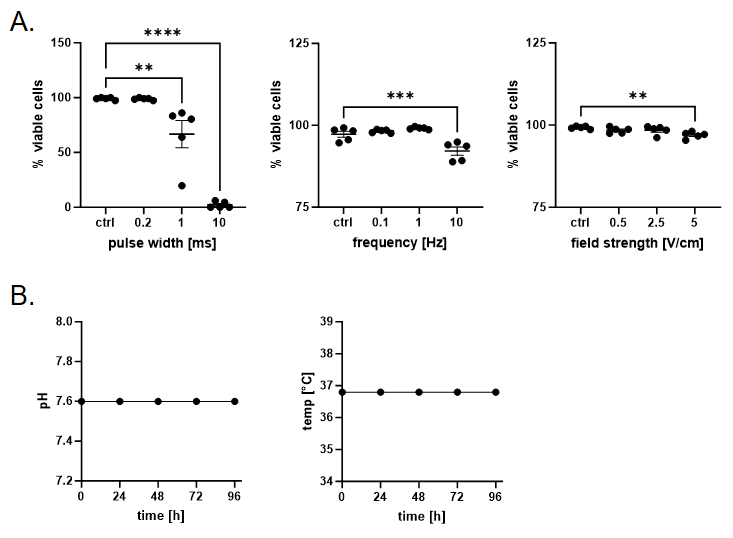


***Figure S1****. A) HUVEC viability following electrical stimulation; b) measured media pH and temperature changes over the course of 4 days of ESTIM.*


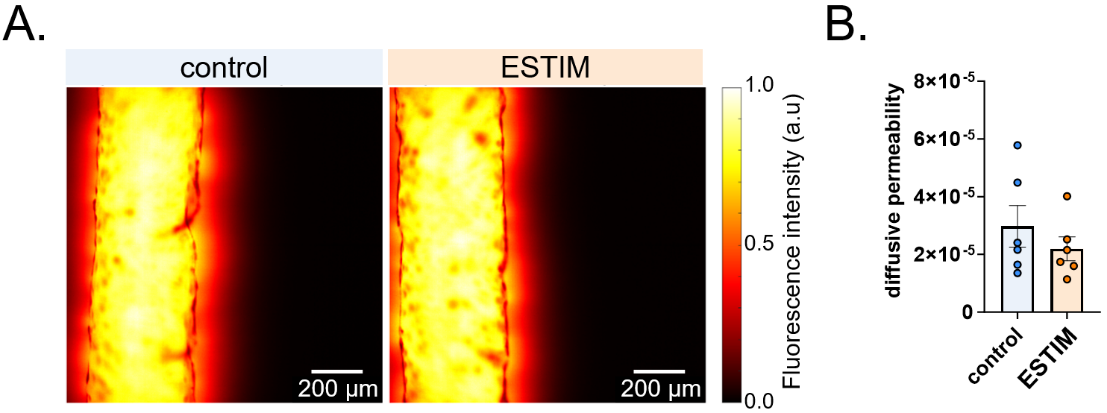


***Figure S2.*** *a) spatial heatmap of FITC-dextran diffusion out of the vessels; b) quantification of diffusive permeability in control vs ESTIM-treated vessels.*


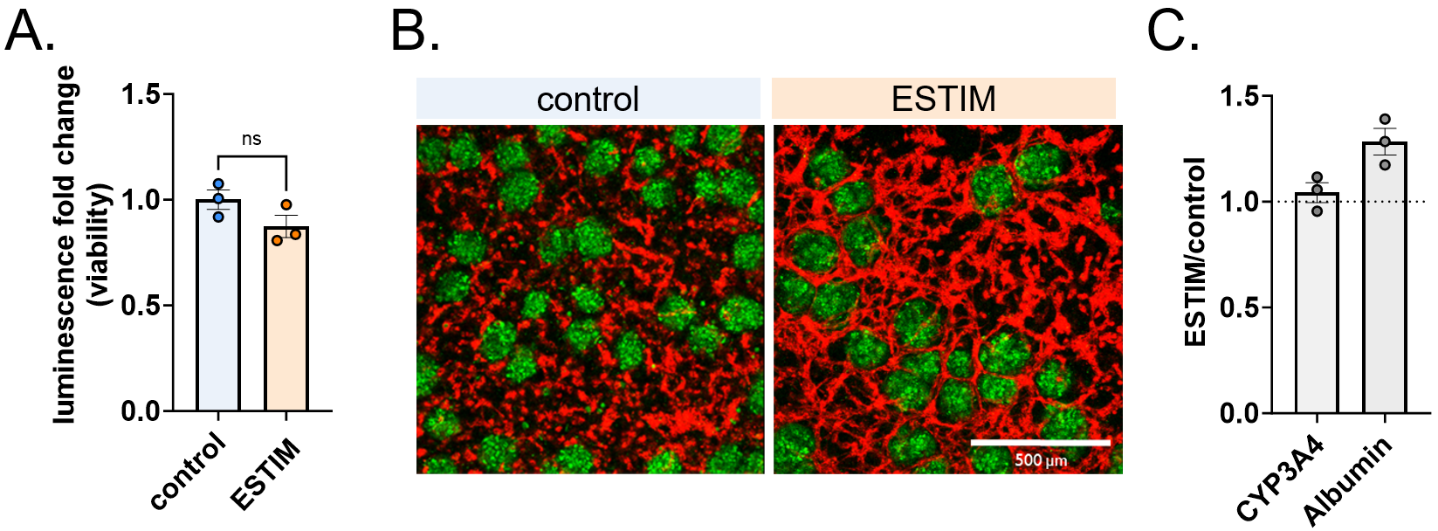
***Figure S3.*** *Hepatocytes are not negatively affected by treatment with ESTIM. a) hepatocyte viability following treatment with ESTIM; b) images of 3D constructs of hepatic spheroids with HUVEC; c) fold change of CYP3A4 activity and albumin secretion of human hepatocytes following ESTIM treatment.*


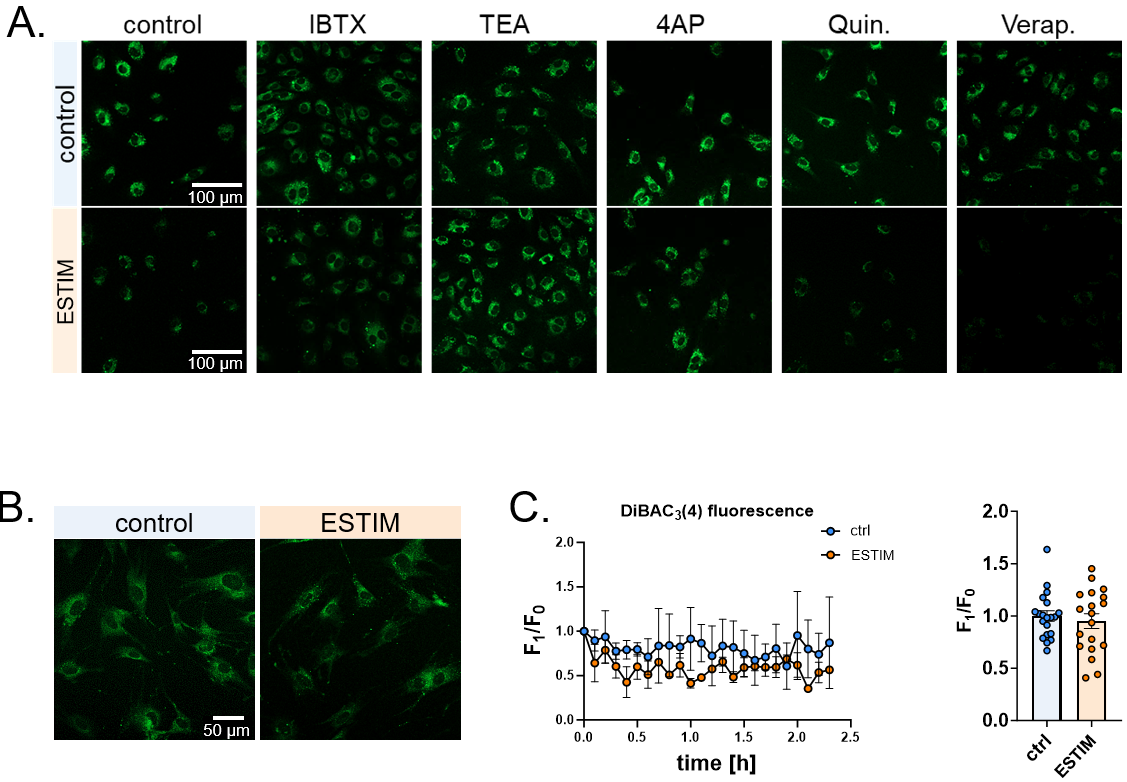


***Figure S4.*** *a) representative fluorescent images of DiBAC_4_(3) (green) in HUVECs following ESTIM treatment in the presence of each of the inhibitors; b) fluorescent images of DiBAC4(3) (green) in NHDFs following 2 h exposure to ESTIM; c) quantification of changes in DiBAC_4_(3) fluorescence in NHDFs over 2 hours of exposure to ESTIM time and quantification of fluorescence changes in 20 individual cells at the 2 h timepoint.*


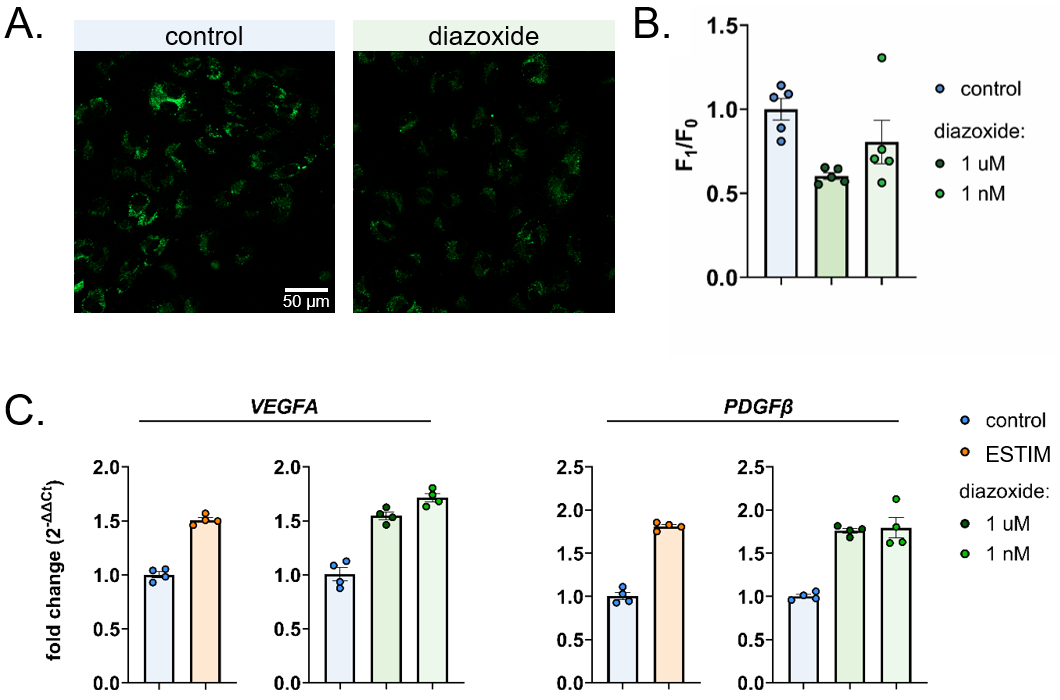


***Figure S5****. Chemical hyperpolarization induces pro-vascular responses in endothelial cells. a) fluorescent images of DiBAC_4_(3)-stained HUVECs (green) following diazoxide treatment; b) quantification of DiBAC_4_(3) fluorescence changes; c) gene expression comparison changes of HUVECs treated with ESTIM vs. diazoxide.*


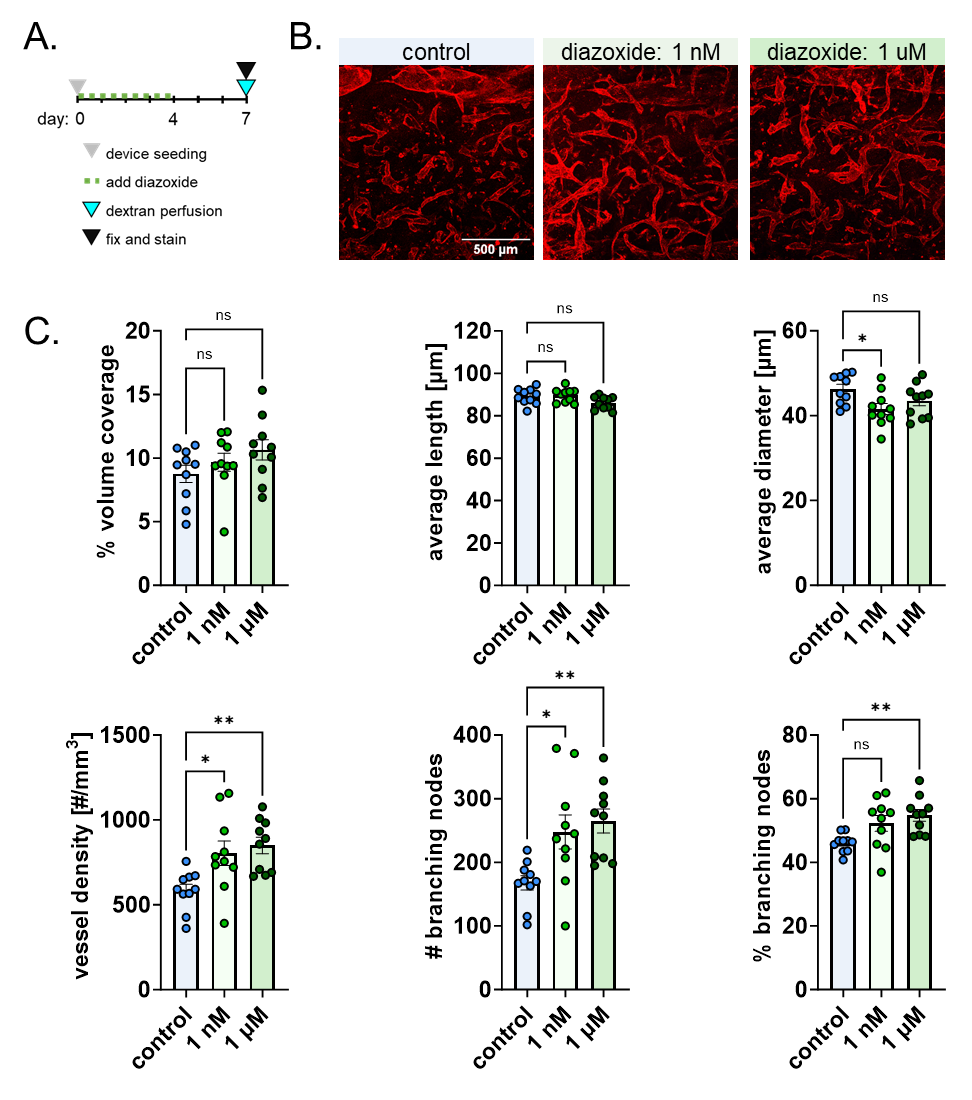


***Figure S6****. Chemically hyperpolarized cultures form denser vasculature. a) experimental timeline; b) fluorescent images of UEA I lectin-stained HUVECs (red) in the microfluidic device following 4-days of diazoxide treatment at 7 day timepoint; c) quantification of network characteristics, ordinary one-way ANOVA, ns not significant, * p<0.05, ** p<0.01.*

***Supplemental Tables***

| ***Table S1.*** *Primer sequences.* | | |
| --- | --- | --- |
| Gene name | Forward | Reverse |
| *GAPDH* | GTCTCCTCTGACTTCAACAGCG | ACCACCCTGTTGCTGTAGCCAA |
| *VEGFA* | TTGCCTTGCTGCTCTACCTCCA | GATGGCAGTAGCTGCGCTGATA |
| *ANG-2* | ATTCAGCGACGTGAGGATGGCA | GCACATAGCGTTGCTGATTAGTC |
| *PLGF* | GGCGATGAGAATCTGCACTGTG | ATTCGCAGCGAACGTGCTGAGA |
| *VIM* | AGGCAAAGCAGGAGTCCACTGA | ATCTGGCGTTCCAGGGACTCAT |
| *PDGFβ* | GAGATGCTGAGTGACCACTCGA | GTCATGTTCAGGTCCAACTCGG |
